# Enhanced IgG4 production by follicular helper 2 T cells and the involvement of follicular helper 1 T cells in the pathogenesis of IgG4-related disease

**DOI:** 10.1186/s13075-016-1064-4

**Published:** 2016-07-13

**Authors:** Mitsuhiro Akiyama, Hidekata Yasuoka, Kunihiro Yamaoka, Katsuya Suzuki, Yuko Kaneko, Harumi Kondo, Yoshiaki Kassai, Keiko Koga, Takahiro Miyazaki, Rimpei Morita, Akihiko Yoshimura, Tsutomu Takeuchi

**Affiliations:** Division of Rheumatology, Department of Internal Medicine, Keio University School of Medicine, Tokyo, Japan; Inflammation Drug Discovery Unit, Pharmaceutical Research Division, Takeda Pharmaceutical Company Limited, Kanagawa, Japan; Department of Microbiology and Immunology, Keio University School of Medicine, Tokyo, Japan

**Keywords:** IgG4-related disease, Follicular helper T cells, B cells, Disease activity

## Abstract

**Background:**

The aim of this study was to elucidate the function of circulating follicular helper T (Tfh) cell subsets in helping B cells in patients with active, untreated IgG4-related disease (IgG4-RD) and determine their relationship with disease activity.

**Methods:**

Seventeen consecutive patients with active, untreated IgG4-RD, 20 with primary Sjögren syndrome (pSS), 5 with multicentric Castleman’s disease (MCD), and 12 healthy controls (HC) were enrolled. Tfh cell subset function was evaluated by co-culture with naïve B cells in vitro. Activated Tfh cell subsets were defined as a CCR7^low^PD-1^high^ subset among Tfh cell subsets. Disease activity was evaluated by IgG4-RD responder index (IgG4-RD RI) score.

**Results:**

The number of Tfh2 cells was significantly higher in IgG4-RD compared to pSS, MCD, or HC, and correlated with serum IgG4 level or the number of plasmablasts. In vitro, Tfh2 cells more efficiently induced the differentiation of naïve B cells into plasmablasts compared to Tfh1 or Tfh17 cells. Of note, while IgG production in culture supernatants of Tfh2 cells was comparable between IgG4-RD and HC, IgG4 production was significantly higher with Tfh2 cells from patients with IgG4-RD than in those from HC. Accordingly, the IgG4:IgG ratio in culture supernatants was also significantly higher with Tfh2 cells from IgG4-RD compared to HC. Moreover, the number of activated Tfh2 cells was higher in IgG4-RD compared to pSS, MCD, or HC, and strongly correlated with IgG4-RD RI score in the baseline active phase. Particularly, the number of activated Tfh2 cells was associated with the number of affected organs and serum IgG4 level. Importantly, the number of activated Tfh2 cells was decreased after glucocorticoid treatment and paralleled disease improvement. Moreover, the number of activated Tfh1 cells was also increased in IgG4-RD compared to pSS, MCD, or HC, correlating with IgG4-RD RI score, but not with serum IgG4 level.

**Conclusions:**

Tfh2 cells, but not Tfh1 or Tfh17 cells, induce the differentiation of naïve B cells into plasmablasts and enhanced production of IgG4 in patients with active, untreated IgG4-RD. Furthermore, activated Tfh2 cells reflect disease activity, suggesting the involvement of this T cell subset in the pathogenesis of IgG4-RD. Interestingly, the number of activated Tfh1 cells was also increased in IgG4-RD, correlating with disease activity but not with serum IgG4 level, suggesting the involvement of Tfh1 cells but not in the process of IgG4 production in patients with IgG4-RD.

**Electronic supplementary material:**

The online version of this article (doi:10.1186/s13075-016-1064-4) contains supplementary material, which is available to authorized users.

## Background

IgG4-related disease (IgG4-RD) is a new disease entity characterized by elevated serum IgG4 and increased infiltration of IgG4^+^ plasma cells in lesions, such as in the salivary and lacrimal glands, pancreas, bile duct, lung, kidney, aorta, retroperitoneum, and lymph nodes [1–5]. IgG4-related dacryoadenitis and sialoadenitis, so-called Mikulicz disease, was originally considered a subtype of primary Sjögren syndrome (pSS) based on the similarity of organ involvement. However, Mikulicz disease was recently recognized as a subtype of IgG4-RD due to high serum IgG4 concentrations and the infiltration of IgG4^+^ plasma cells in glandular tissues [6]. Elevated serum IgG4 and IgG4^+^plasma cell infiltration in lesions have also been reported in multicentric Castleman’s disease (MCD), a rare and poorly understood lymphoproliferative disorder [7–12]. As they satisfy the diagnostic criteria of IgG4-RD, the differentiation between IgG4-RD and MCD on the basis of serum IgG4 level or the histologic finding alone is recognized to be difficult [7–12].

Interestingly, recent reports have shown that the number of circulating plasmablasts positively correlates with disease activity in IgG4-RD [13, 14]. The expansion of plasmablasts is accompanied by enhanced somatic mutation and the emergence of distinct plasmablast clones related to relapse of IgG4-RD, suggesting the linkage of disease pathogenesis to *de novo* recruitment of naïve B cells into T-cell-dependent responses [15]. Collaboration of follicular helper T (Tfh) cells and B cells at the germinal center plays a major role in antibody production, immunoglobulin-isotype switching, affinity maturation, and plasmablast and plasma-cell genesis [16, 17]. Indeed, in IgG4-RD, germinal centers are often observed within affected organs [18] and are presumably the source of plasmablasts. In general, bona fide Tfh cells have initially been identified in secondary lymphoid organs but their counterparts and subsets (Tfh1, Tfh2, or Tfh17 cells) have only been recognized in peripheral blood [19]. We previously reported that the number of circulating Tfh2 cells is increased in IgG4-RD in correlation with elevated serum IgG4 and the number of plasmablasts, suggesting the important role of Tfh2 cells in IgG4-RD pathogenesis [20, 21]. However, the question of whether Tfh2 cells actually induce B cells to differentiate into plasmablasts and to produce IgG4 in patients with IgG4-RD remains unanswered. Functional analysis by in vitro assay is thus desired.

Simpson et al. initially described the expansion of circulating Tfh cells in patients with systemic lupus erythematosus that is the prototype of human autoimmune disease [22]. Recently, circulating Tfh cells have been reported to be a valuable biomarker for the monitoring of dysregulated antibody responses and disease activity in autoimmune diseases [22–25]. Defining therapeutic targets for IgG4-RD requires a clear understanding of the pathogenic pathways and corresponding biomarkers of disease activity. Recent reports have shown that detection of the CCR7^low^PD-1^high^ subset, the “activated Tfh cells” in circulation, is a useful tool in monitoring the activation status of Tfh cells in autoimmunity, human immunodeficiency virus infection, and vaccination [22–26]. Indeed, a high percentage of activated Tfh cells was observed in Tfh-biased autoimmune sanroque mice and patients with systemic lupus erythematosus with high autoantibody titers and severe disease activity [26]. These observations suggest that circulating activated Tfh cells may link to disease activity in Tfh-biased diseases. To date, however, this question is uncertain in patients with IgG4-RD.

Thus, herein we sought to investigate the functional role of Tfh cell subsets in helping B cells, and assessed the expansion of activated Tfh cell subsets for correlation with disease activity, in the blood of patients with active, untreated IgG4-RD, and comparing this to patients with pSS or MCD and to healthy controls (HCs).

## Methods

### Patients

Seventeen consecutive patients with active, untreated IgG4-RD (nine women and eight men, mean age 59.5 ± 3.1 years), who were referred to our institution were enrolled as the test subjects in this study. In total 20 patients with pSS (seventeen17 women and 3 men, mean age 61.8 ± 3.0 years), 5 patients with MCD (4 women and 1 man, mean age 52.8 ± 2.9 years), and 12 HC (6 women and 6 men, mean age 48.4 ± 2.6 years) were included. All patients with IgG4-RD fulfilled the 2011 comprehensive IgG4-RD diagnostic criteria [27]. Furthermore, none of the patients were receiving glucocorticoid (GC) treatment at the time of enrollment. All patients with pSS fulfilled the 2002 American-European Consensus Group criteria [28], and were confirmed not to have IgG4-RD. All patients with MCD were diagnosed by pathological confirmation of the plasma-cell or the mixed-type variant based on a previous report [29]. No patient with MCD was infected with human immunodeficiency virus. Demographic features and results of laboratory tests were collected from medical records. Disease activity in IgG4-RD was defined based on the IgG4-RD responder index (IgG4-RD RI) score [30], with active disease indicated by a score >3, as previously described [20]. HCs were confirmed to have no autoimmune diseases, allergic disorders, malignancies, or infections. This study was approved by the ethics committee of our institution and carried out in accordance with the Declaration of Helsinki and Good Clinical Practice. Written informed consent was obtained from all the patients and HCs.

### Detection of Tfh cells and their subsets with flow cytometry

Immunophenotyping of lymphocytes was performed by multicolor flow cytometry. Heparinized whole blood samples were immediately stained for 15 minutes with the following antibodies: CD3-APC/Cy7 (UCHT, BioLegend; San Diego, CA, USA), CD4-HorizonV500 (RPA-T4, BD Biosciences; San Jose, CA, USA), CD45RA-BV421 (HI-100, BioLegend), CXCR5-PerCP/Cy5.5 (J252D4, BioLegend), CXCR3-APC (1C6, BD Biosciences), CCR6-PE (11A9, BD Biosciences), CCR7-PE/Cy7 (G043H7, BioLegend), PD-1-FITC (EH12.2H7, BioLegend), CD19-BV421 (HIB19, BioLegend), CD20-APC (2H7, BioLegend), CD27-APC/Cy7 (O323, BioLegend), and CD38-FITC (HIT2, BioLegend). Circulating Tfh cells were defined as CD3^+^CD4^+^CD45RA^-^CXCR5^+^ cells, and Tfh1, Tfh2, or Tfh17 cell subsets as CXCR3^+^CCR6^-^ cells, CXCR3^-^CCR6^-^ cells, or CXCR3^-^CCR6^+^ cells among Tfh cells, respectively [19, 20]. Activated Tfh cells were defined as the CCR7^low^PD-1^high^ cells among Tfh cells [26]. CD19^+^CD20^-^CD27^+^CD38^+^ cells were defined as plasmablasts. Red blood cells were lysed with FACS Lysing Solution (BD Biosciences). All samples were analyzed with a FACS Aria III (BD Biosciences), and data were analyzed with FlowJo v.7.6.4 Software (Tree Star, Stanford University, CA, USA). Proportions of lymphocyte subsets were determined by the combination of surface marker staining, with exclusion of doublets by forward and side scatter.

### Sorting of Tfh cell subsets and naïve B cells from peripheral blood mononuclear cells (PBMCs)

PBMCs were separated by gradient centrifugation with a Lymphoprep (Axis-Shield; Oslo, Norway) according to the manufacturer’s instructions. CD4^+^ T cells and CD19^+^ B cells were enriched from PBMCs using a CD4^+^ T cell isolation kit and CD19^+^ B cell isolation kit, respectively (Miltenyi Biotec; Bergisch Gladbach, Germany) according to the manufacturer’s instructions. For sorting of Tfh cell subsets, enriched CD4^+^ T cells were stained with anti-CCR6-PE (11A9, BD Biosciences), anti-CXCR3-APC (1C6, BD Biosciences), anti-CXCR5-PerCP/Cy5.5 (J252D4, BioLegend), anti-CD45RA-BV421 (HI-100, BioLegend), and anti-CD4-HorizonV500 (RPA-T4, BD Biosciences). Tfh cell subsets were sorted from CD4^+^ T cells as Tfh2 (CXCR3^-^CCR6^-^), Tfh1 (CXCR3^+^CCR6^-^), or Tfh17 (CXCR3^-^CCR6^+^) cells among CXCR5^+^CD45RA^-^CD4^+^ T cells [19]. For sorting of naïve B cells, enriched CD19^+^ B cells were stained with anti-IgD-PE (IA6-1, Biolegend) and anti-CD27-APC/Cy7 (O323, BioLegend). Naïve B cells were sorted from CD19^+^ B cells as IgD^+^CD27^-^CD19^+^ cells. Tfh cell subsets and naïve B cell sorting was conducted with a FACS Aria III (BD Biosciences) according to the manufacturer’s instructions. Cell purity of naïve B cells determined by flow cytometry was greater than 98 % and cell purity was greater than 93 % for Tfh cell subsets.

### In vitro induction of B-cell maturation by Tfh cell subsets

Autologous naïve B cells (1 × 10^4^ cells) were co-cultured with sorted Tfh cell subsets (1 × 10^4^ cells) in the presence of endotoxin-reduced staphylococcal enterotoxin B (SEB) (Sigma-Aldrich; Steinheim, Germany) (1 μg/ml) in Roswell Park Memorial Institute (RPMI) 1640 medium supplemented with 10 % heat-inactivated fetal bovine serum in 96-well round-bottom plates. At day 7, cells were collected and stained with fluorescent-conjugated antibodies for flow cytometry, and plasmablasts (CD19^+^CD27^+^CD38^+^ cells) were detected by flow cytometry. Concentration of IgG, IgG4 or IL-4 in each culture supernatant was measured at day 7 using cytometric bead array (BD Biosciences) according to the manufacturer’s instructions using an LSRFortessa X-20 (BD Biosciences).

### Statistical analysis

Continuous variables are shown as mean ± SEM. Multiple group comparisons were analyzed using the Kruskal-Wallis test. When results were significant (*P* < 0.05), respective group-wise comparisons were performed using the Mann-Whitney *U* test. Differences between pre- and post-treatment data were assessed using the Wilcoxon signed-rank test. Correlations between two groups were analyzed using Spearman’s correlation coefficient. Statistical significance was determined using GraphPad Prism software V.6.0 (GraphPad software; San Diego, CA, USA), with *P* < 0.05 considered significant.

## Results

### Clinical characteristics and demographics

The clinical characteristics of the 17 patients with IgG4-RD are described in Table 1. The mean concentrations of serum IgG4, IgE and soluble IL-2 receptor (sIL-2R) were 481.4 ± 106.4 mg/dL (normal <105 mg/dL), 530.5 ± 165.1 IU/mL (normal <170 IU/mL), and 500.1 ± 104.6 U/mL (normal <500 U/mL), respectively. C-reactive protein (CRP) was within the normal range (0.06 ± 0.02 mg/dL; normal <0.35 mg/dL). Of the 17 patients with IgG4-RD, 11 (64.7 %) had concomitant allergic disorders. Frequent sites of organ involvement included the submandibular glands (13 cases, 76.5 %) and lacrimal glands (12 cases, 70.6 %). The average IgG4-RD RI score was 11.3 ± 1.4.

**Table 1:** Clinical and laboratory characteristics of patients with IgG4-RD

Serum IgG (normal <1700 mg/dL)^a^	1853.8 ± 191.2
Serum IgG4 (normal <105 mg/dL)^a^	481.4 ± 106.4
Serum IgE (normal <170 IU/mL)^a^	530.5 ± 165.1
Soluble IL-2 receptor (normal <500 U/mL) (n = 14)	500.1 ± 104.6
Eosinophils in lymphocytes, % (normal <6 %) (n = 16)	7.0 ± 1.6
LDH (normal <220 IU/L)^a^	165.9 ± 6.9
CRP (normal <0.35 mg/dL)^a^	0.06 ± 0.02
IgG4-RD RI score^a^	11.3 ± 1.4
Allergic history	11 (64.7 %)
Organs involved	
Submandibular gland	13 (76.5 %)
Lacrimal gland	12 (70.6 %)
Lymph node	6 (35.3 %)
Parotid gland	5 (29.4 %)
Kidney	5 (29.4 %)
Lung	5 (29.4 %)
Pancreas	3 (17.6 %)
Aorta	2 (11.8 %)
Retroperitoneal fibrosis	2 (11.8 %)
Skin	1 (5.9 %)

^a^Mean ± SEM (n = 17). *IgG4-RD* IgG4-related disease, *IL-2* interleukin-2, *LDH* lactate dehydrogenase, *CRP* C-reactive protein, *IgG4-RD RI score* IgG4-related disease responder index score

### Circulating Tfh2 cells and their correlation with clinical parameters in IgG4-RD

**Fig. 1:**
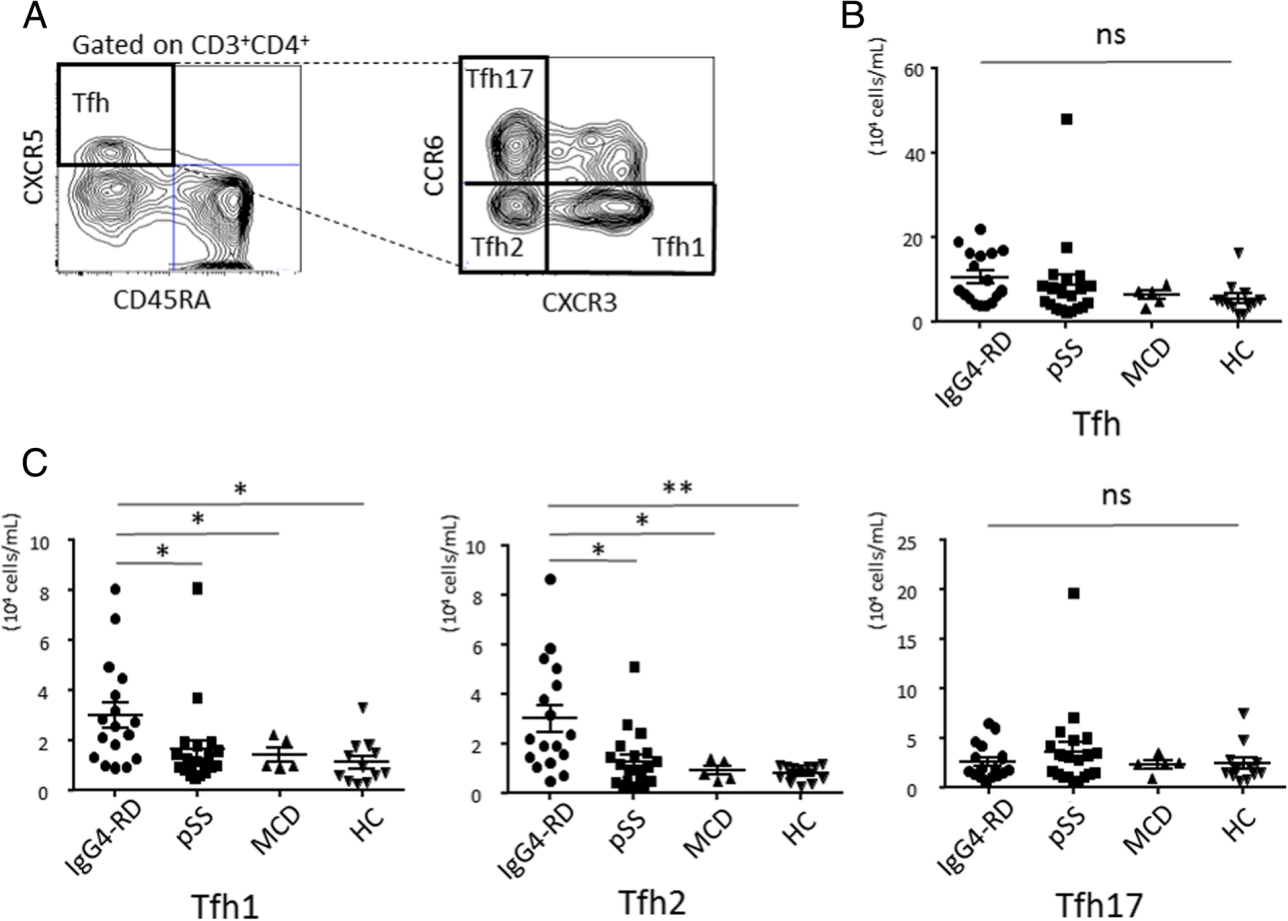
Flow cytometric analysis of the number of circulating follicular helper T (*Tfh*) cells and Tfh cell subsets. Representative flow cytometric analyses of Tfh cells and Tfh cell subsets (**a**). The absolute number of Tfh cells (**b**) and Tfh cell subsets (**c**) from patients with IgG4-related disease (*IgG4-RD*) (n = 17), primary Sjögren’s syndrome (*pSS*) (n = 20), multicentric Castleman’s disease (*MCD*) (n = 5), and healthy controls (*HC*) (n = 12). *P < 0.05; **P < 0.0001 for analysis using the Kruskal-Wallis test, followed by group-wise comparisons using the Mann-Whitney *U* test. *ns* not significant

**Fig. 2:**
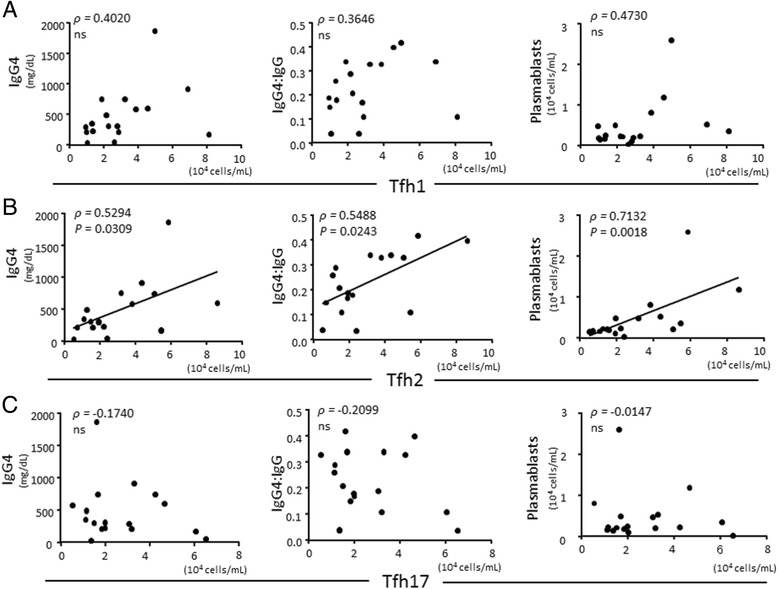
Correlation between the number of follicular helper T (*Tfh*) cell subsets and serum IgG4 level, or IgG4:IgG ratio, or the number of plasmablasts in patients with IgG4-related disease (*IgG4-RD*). Tfh1 cells (**a**), Tfh2 cells (**b**), Tfh17 cells (**c**). N = 17. Correlation was tested by calculating Spearman’s correlation coefficient. *ns* not significant

We previously reported that the number of circulating Tfh2 cells in patients with IgG4-RD was increased compared to patients with pSS or allergic rhinitis and HCs, and correlated with serum IgG4 level, IgG4:IgG ratio, and the number of plasmablasts [20]. MCD is a rare lymphoproliferative disorder which is accompanied by elevated serum IgG4; it was also elevated in the present study, and was not significantly different in IgG4-RD (179.8 ± 67.9 mg/dL vs 481.4 ± 106.4 mg/dL, *P* = 0.1400). As the pathogenesis of MCD is considered to be different from that of IgG4-RD, we hypothesized that the number of circulating Tfh2 cells can help to discriminate MCD from IgG4-RD. As illustrated in Fig. 1a, we detected Tfh cells and their subsets. The number of Tfh cells was not statistically different among groups (IgG4-RD, 104402 ± 14663 cells/mL; pSS, 87032 ± 2234 cells/mL; MCD, 61788 ± 9908 cells/mL; HC, 54917 ± 11072 cells/mL, *P* = 0.1096) (Fig. 1b), while the percentage of Tfh cells was significantly increased in IgG4-RD (16.66 ± 1.831 %, *P* = 0.0177), pSS (21.65 ± 1.989 %, *P* < 0.0001), MCD (17.11 ± 4.054 %, *P* = 0.0388) compared to HC (9.777 ± 1.006 %) (Additional file 1: Figure S1A). Regarding Tfh cell subsets, the number of Tfh2 cells was significantly increased in patients with IgG4-RD (30035 ± 5369 cells/mL) compared to patients with pSS (12646 ± 2584 cells/mL, *P* = 0.0025) or MCD (9166 ± 1809 cells/mL, *P* = 0.0110), and to HCs (8026 ± 949.0 cells/mL, *P* < 0.0001) (Fig. 1c). In addition, the number of Tfh1 cells was also significantly increased in patients with IgG4-RD (30383 ± 5018 cells/mL) compared to patients with pSS (16537 ± 3743 cells/mL, *P* = 0.0043) or MCD (14512 ± 2835 cells/mL, *P* = 0.0450), and to HCs (11461 ± 2549 cells/mL, *P* = 0.0016) (Fig. 1c). On the other hand, the number of Tfh17 cells was not different among groups (IgG4-RD, 26,859 ± 4272 cells/mL; pSS, 37,040 ± 9319 cells/mL; MCD, 23,829 ± 4089 cells/mL; HC, 24410 ± 5596 cells/mL, *P* = 0.8367) (Fig. 1c). We also examined the relationship between the number of Tfh cell subsets and the level of IgG4, or the number of plasmablasts, focusing on patients with IgG4-RD. Importantly, we confirmed that increased number of Tfh2 cells positively correlated with serum level of IgG4, IgG4:IgG ratio, and the number of plasmablasts in patients with active, untreated IgG4-RD (Fig. 2b), as previously reported [20]. On the other hand, there was no correlation among the number of Tfh1 or Tfh17 cells and serum level of IgG4 or IgG4:IgG ratio or the number of plasmablasts (Fig. 2a and 2c).

### Induction by Tfh2 cells of differentiation of naïve B cells into plasmablasts

**Fig. 3:**
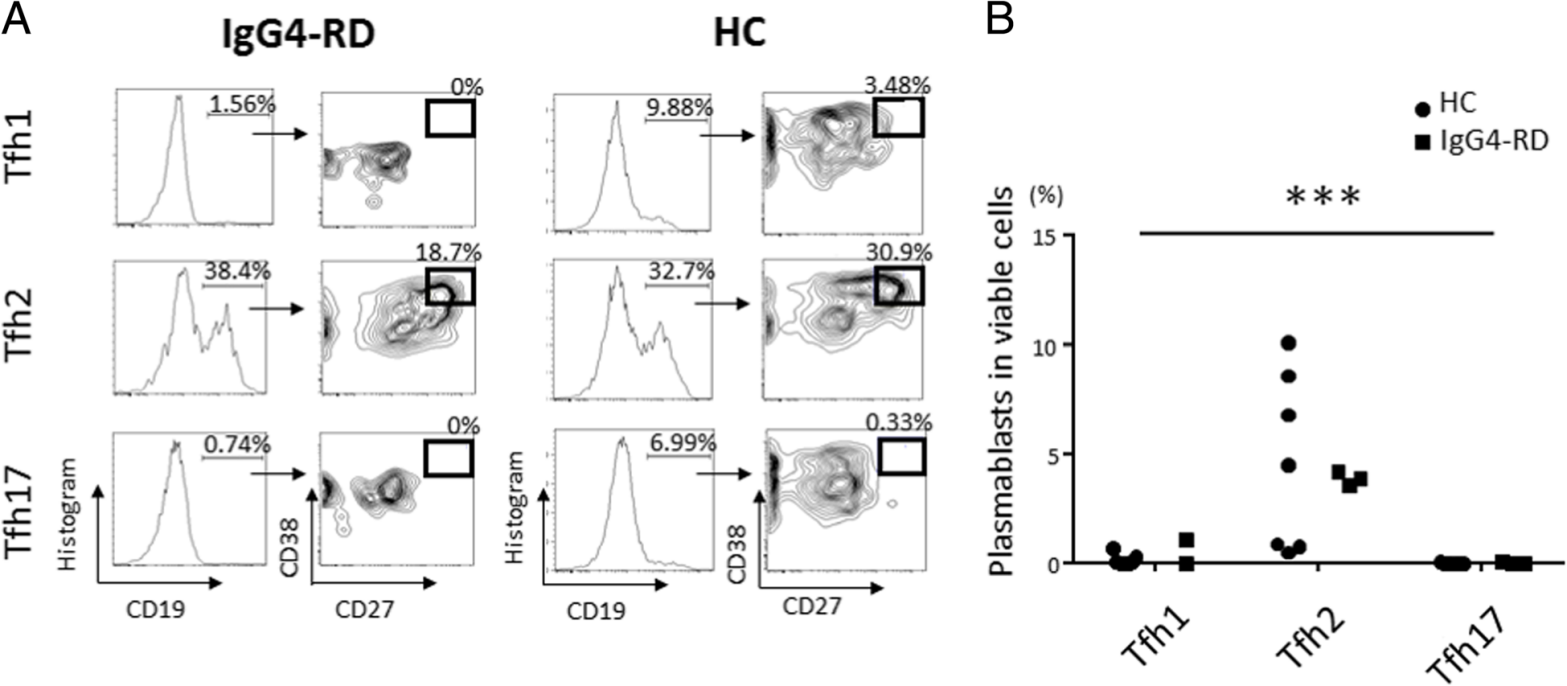
Induction of the differentiation of naïve B cells into plasmablasts by circulating follicular helper T (*Tfh*) cell subsets. Sorted Tfh subsets (*Tfh1*, *Tfh2*, and *Tfh17* cells) from patients with IgG4-related disease (*IgG4-RD*) or healthy controls (*HC*) were co-cultured with autologous naive B cells for 7 days and analyzed by flow cytometry. Representatives of the CD19^+^CD27^+^CD38^+^ plasmablast population (**a**). Percentage of plasmablasts at day 7 induced by different Tfh cell subsets from patients with IgG4-RD and HC (**b**). ***P < 0.0001 for analysis using the Kruskal-Wallis test

Next, we hypothesized that Tfh2 cells may play a role in the process of T-B interaction and contribute to the induction of naïve B cell differentiation into plasmablasts in patients with IgG4-RD. To examine our hypothesis, we sorted Tfh-cell subsets from PBMCs of patients with active, untreated IgG4-RD or HCs, and co-cultured them with naïve B cells. Importantly, compared to Tfh1 and Tfh17 cells, Tfh2 cells more efficiently induced naïve B cells to differentiate into plasmablasts in both patients with IgG4-RD and HCs (Fig. 3a and 3b).

### Induction by Tfh2 cells of IgG4 production

**Fig. 4:**
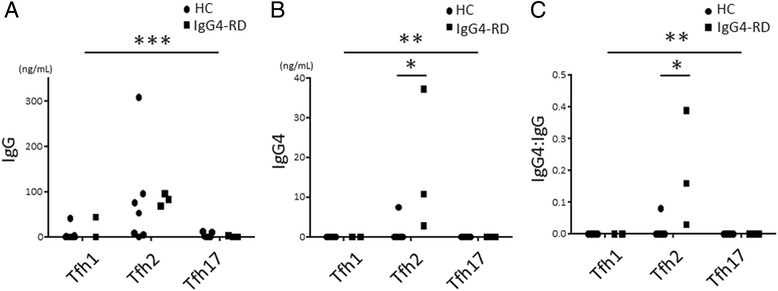
Induction of IgG4 production by circulating follicular helper T (*Tfh*) cell subsets. Sorted Tfh cell subsets (*Tfh1*, *Tfh2*, and *Tfh17* cells) from patients with IgG4-related disease (*IgG4-RD*) or healthy controls (HC) were co-cultured with autologous naive B cells for 7 days, and IgG (**a**) and IgG4 (**b**) in the supernatant was measured by cytometric bead array. The IgG4:IgG ratio was calculated (**c**). **P < 0.05; ***P < 0.0001 for analysis using the Kruskal-Wallis test; *P < 0.05 for analysis using the Mann-Whitney *U* test

We then analyzed IgG and IgG4 production in culture supernatants to examine whether Tfh cell subsets could induce IgG4 production. Importantly, compared to Tfh1 and Tfh17 cells, Tfh2 cells more efficiently induced the production of IgG in both patients with IgG4-RD and HCs (Fig. 4a). Of note, while Tfh2 cells induced comparable amounts of IgG in patients with IgG4-RD and HCs (Fig. 4a), IgG4 production was significantly higher with Tfh2 cells from patients with active, untreated IgG4-RD than with those from HCs (Fig. 4b). When we examined the IgG4:IgG ratio in the culture supernatants, the IgG4:IgG ratio was also significantly higher with Tfh2 cells from patients with IgG4-RD than with those from HCs (Fig. 4c). IL-4 was significantly higher in culture supernatants with Tfh2 cells (1.521 ± 0.9290 pg/mL) compared to those with Tfh1 cells (0.1856 ± 0.1476 pg/mL) or Tfh17 cells (0.0 ± 0.0 pg/mL) (*P* = 0.0093), however, there was no difference between patients with IgG4-RD and HCs (0.9367 ± 0.5741 pg/mL vs 1.771 ± 1.330 pg/mL, *P* = 0.7038).

### Activated Tfh1 and Tfh2 cells in IgG4-RD

**Fig. 5:**
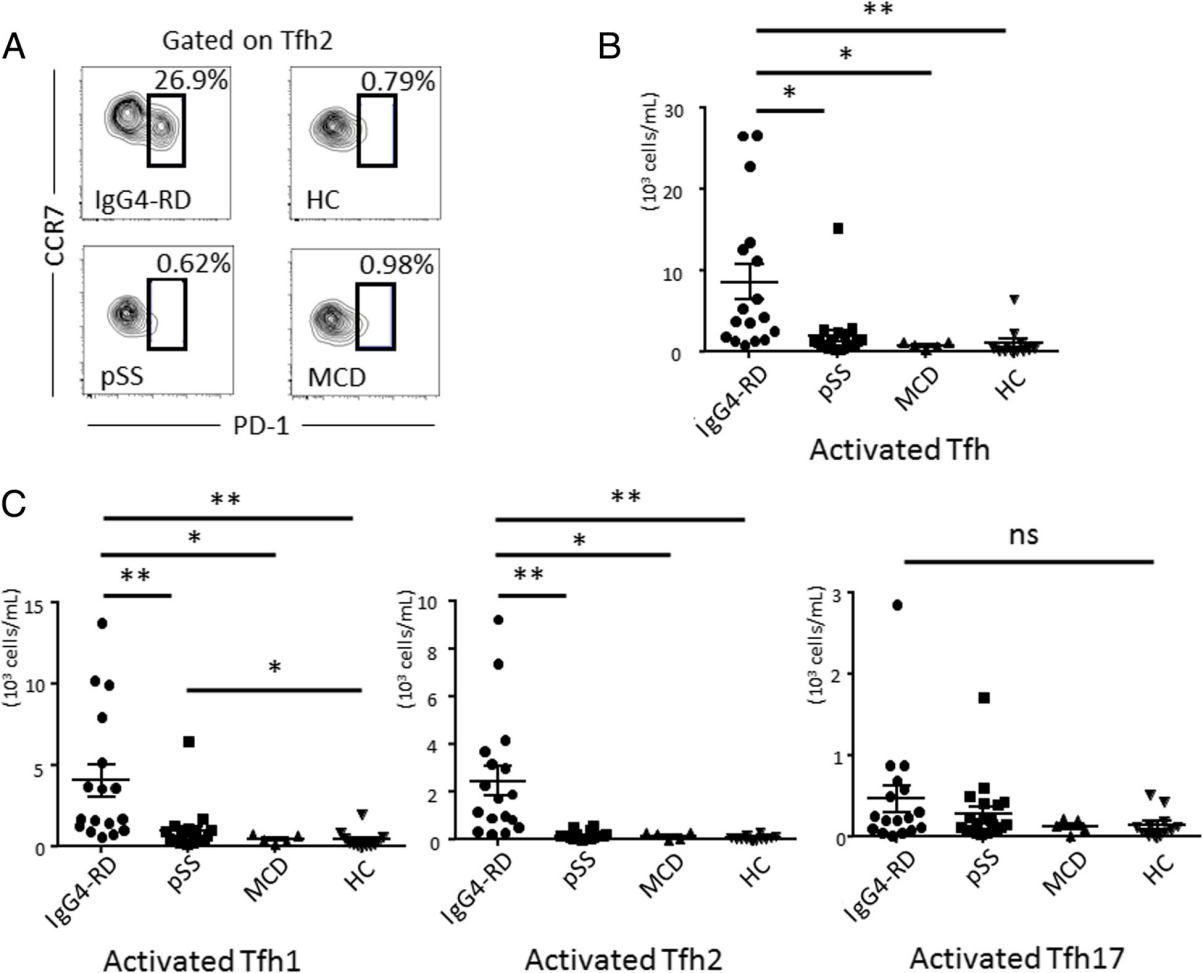
Flow cytometric analysis of the number of circulating activated follicular helper T (*Tfh*) cells and activated Tfh cell subsets. Representative flow cytometric analysis of activated Tfh2 cells (**a**). The absolute number of activated Tfh cells (**b**) and activated Tfh cell subsets (**c**) from patients with IgG4-related disease (*IgG4-RD*) (n = 17), primary Sjögren’s syndrome (*pSS*) (n = 20), multicentric Castleman’s disease (*MCD*) (n = 5) or from healthy controls (*HC*) (n = 12). *P < 0.05; **P < 0.0001 for analysis using the Kruskal-Wallis test, followed by group-wise comparisons using the Mann-Whitney *U* test. *ns* not significant

We further investigated the proportion of activated Tfh cells detected by the expression of CCR7 and PD-1 in active, untreated IgG4-RD (Fig. 5a). The number of activated Tfh cells was increased in patients with IgG4-RD (8648 ± 2164 cells/mL) compared to patients with pSS (1926 ± 722.7 cells/mL, *P* = 0.0001) or MCD (870.4 ± 171.4 cells/mL, *P* = 0.0003), or to HCs (1074 ± 5204 cells/mL, *P* < 0.0001) (Fig. 5b). Along these lines, the percentage of activated Tfh cells was also significantly increased in patients with IgG4-RD (8.067 ± 1.753 %) compared to patients with pSS (2.259 ± 0.3374 %, *P* < 0.0001) or MCD (1.548 ± 0.4033 %, *P* = 0.0014), or to HCs (1.441 ± 0.3178 %, *P* < 0.0001) (Additional file 1: Figure S1B). Interestingly, when we analyzed the activated Tfh cell subsets, the number of activated Tfh2 cells was significantly increased in patients with IgG4-RD (2465 ± 613.7 cells/mL) compared to patients with pSS (172.3 ± 36.12 cells/mL, *P* < 0.0001) or MCD (170.0 ± 59.84 cells/mL, *P* = 0.0005), or to HC (91.75 ± 22.95 cells/mL, *P* < 0.0001) (Fig. 5c). The number of activated Tfh1 cells was also increased in patients with IgG4-RD (4038 ± 972.7 cells/mL) compared to patients with pSS (924.7 ± 307.4 cells/mL, *P* < 0.0001) or MCD (464.9.0 ± 105.0 cells/mL, *P* = 0.0003) or to HCs (400.8 ± 157.7 cells/mL, *P* < 0.0001) (Fig. 5c), while the number of activated Tfh17 cells was not different among groups (IgG4-RD, 466.7 ± 164.4 cells/mL; pSS, 282.0 ± 837.9 cells/mL; MCD, 125.7 ± 37.29 cells/mL; HC, 139.4 ± 47.28 cells/mL, *P* = 0.1835) (Fig. 5c).

### Activated Tfh1 and Tfh2 cells correlated with sIL-2R in IgG4-RD

**Fig. 6:**
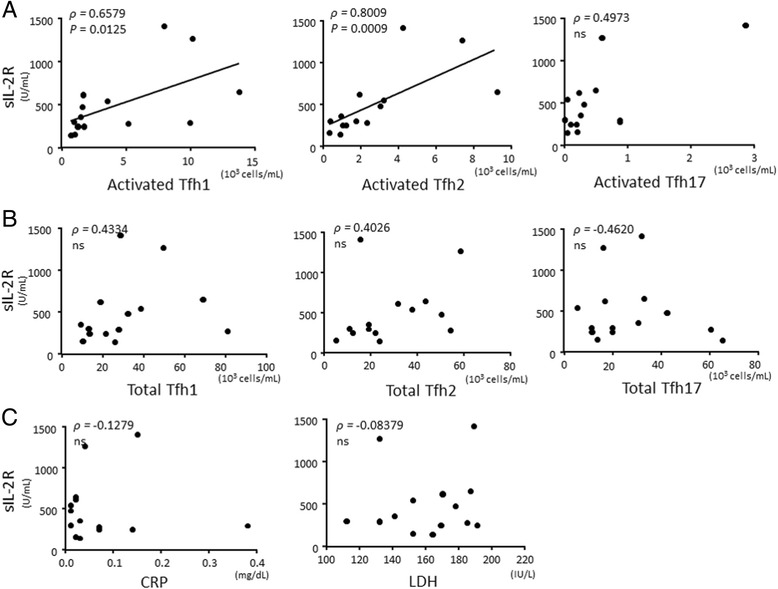
Correlation between the number of activated follicular helper T (*Tfh*) cell subsets and soluble interleukin-2 receptor (*sIL-2R*) in patients with IgG4-related disease. Correlation between sIL-2R and the number of activated Tfh cell subsets (**a**), or total Tfh cell subsets (**b**), and levels of C-reactive protein (*CRP*) and lactate dehydrogenase (*LDH*) (**c**) in patients with IgG4-related disease (*IgG4-RD*) (n = 14). Correlation was tested by calculating Spearman’s correlation coefficient. *ns* not significant

sIL-2R has previously been reported as relatively high in IgG4-RD and correlated with disease activity [31–34]. In general, high sIL-2R is recognized in the activation of lymphocytes, inflammation, and malignancy [34]. Thus, we analyzed correlation between sIL-2R levels and the number of activated Tfh cell subsets or total Tfh cell subsets, serum CRP levels, and lactate dehydrogenase (LDH), in patients who were eligible for measurement of all items in active, untreated IgG4-RD. Interestingly, sIL-2R level was positively correlated with the number of activated Tfh1 cells or activated Tfh2 cells, but not with the number of activated Tfh17 cells, total Tfh1 cells, total Tfh2 cells or total Tfh17 cells (Fig. 6a and 6b). Serum CRP or lactate dehydrogenase (LDH) was not correlated with sIL-2R in patients with IgG4-RD (Fig. 6c).

### Activated Tfh1 and Tfh2 cells correlated with disease activity in IgG4-RD

**Fig. 7:**
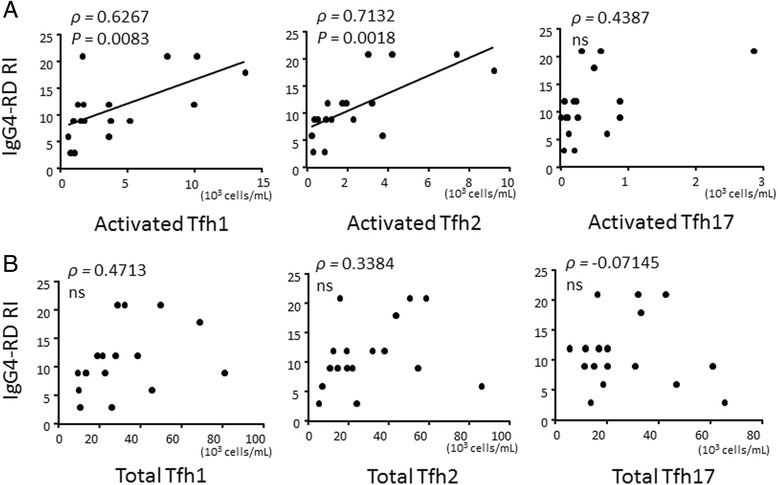
Correlation between the number of activated follicular helper T (*Tfh*) cell subsets and IgG4-related disease responder index (*IgG4-RD RI*) score in patients with IgG4-related disease. Correlation between the number of activated Tfh cell subsets (**a**) or total Tfh cell subsets (**b**) and IgG4-related disease responder index (IgG4-RD RI) score in patients with IgG4-related disease (IgG4-RD) (n = 17). Correlation was tested by calculating Spearman’s correlation coefficient. *ns* not significant

We next assessed whether the expansion of activated Tfh cell subsets was associated with disease activity in IgG4-RD measured by IgG4-RD RI score. Importantly, the baseline IgG4-RD RI score strongly correlated with the number of activated Tfh1 cells or activated Tfh2 cells, but not with the number of activated Tfh17 cells (Fig. 7a). There was no correlation between the IgG4-RD RI score and the number of total Tfh1 cells, total Tfh2 cells, or total Tfh17 cells (Fig. 7b). There was also no correlation between the IgG4-RD RI score and the number of plasmablasts or eosinophils, or serum IgE levels (data not shown).

### Activated Tfh cell subsets and their correlation with the number of affected organs or serum IgG4 levels in IgG4-RD

**Fig. 8:**
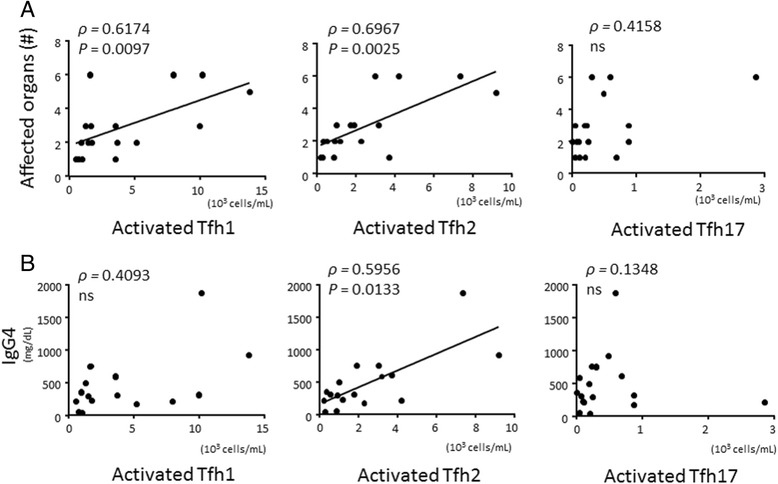
Correlation between the number of activated follicular helper T (*Tfh*) cell subsets and the number of affected organs or serum IgG4 level in patients with IgG4-related disease. Correlation between the number of activated Tfh cell subsets and the number of affected organs (**a**) or serum IgG4 level (**b**) in patients with IgG4-related disease (IgG4-RD) (n = 17). *ns* not significant

We further examined the association between the number of activated Tfh cell subsets and serum IgG4 level, or the number of affected organs as a measure of disease severity. Of note, the number of affected organs positively correlated with the number of activated Tfh1 cells and the number of activated Tfh2 cells, but not with the number of activated Tfh17 cells (Fig. 8a). In line with our in vitro study (Fig. 4), serum IgG4 positively correlated with the number of activated Tfh2 cells, but not with the number of activated Tfh1 cells or activated Tfh17 cells (Fig. 8b).

### Activated Tfh cell subsets following treatment with GC in IgG4-RD

**Fig. 9:**
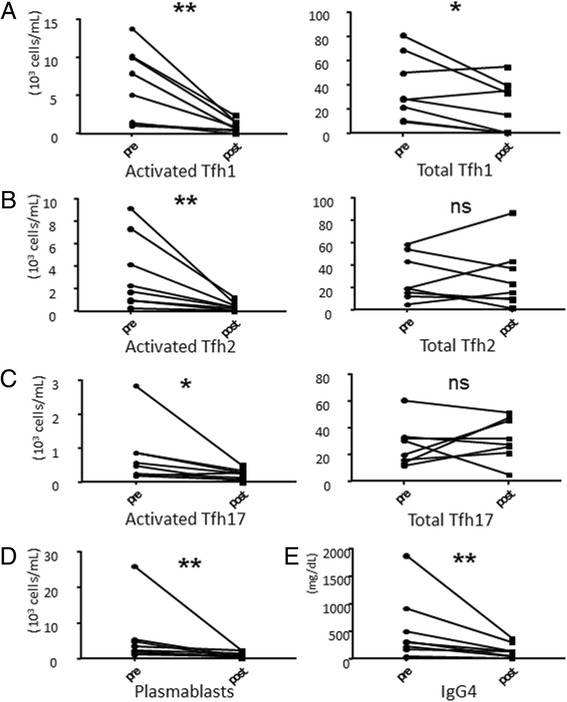
The number of activated follicular helper T (*Tfh*) cell subsets, and total Tfh cell subsets, plasmablasts, and serum IgG4 level after glucocorticoid (GC) treatment in patients with IgG4-related disease. The number of activated and total Tfh1 cells (**a**), Tfh2 cells (**b**), Tfh17 cells (**c**), and plasmablasts (**d**), and level of serum IgG4 (**e**). N = 8. *P < 0.05; **P < 0.01 for analysis using the Wilcoxon signed-rank test. *ns* not significant

We serially evaluated the number of activated Tfh cell subsets in eight patients with IgG4-RD before and after treatment with GC to compare the baseline untreated active phase and an inactive phase 6–24 weeks after treatment initiation. The IgG4-RD RI score was significantly improved after GC treatment in seven patients for whom clinical data were available (11.6 ± 2.1 vs 1.7 ± 0.5, *P* < 0.001). The number of activated Tfh2 cells, activated Tfh1 cells, and activated Tfh17 cells significantly decreased after GC treatment (activated Tfh2 cells, 3372 ± 1162 cells/mL vs 406.9 ± 132.9 cells/mL, *P* = 0.0078, and activated Tfh1 cells, 6332 ± 1716 cells/mL vs 997.2 ± 273.0, *P* = 0.0078, and activated Tfh17 cells, 791.3 ± 310.5 cells/mL vs 230.0 ± 55.91, *P* = 0.0156) (Fig. 9a-c). The number of plasmablasts also decreased (5861 ± 2926 cells/mL vs 1100 ± 312.5 cells/mL, *P* = 0.0078) (Fig. 9d) and the decline in serum IgG4 was significant post treatment (539.6 ± 212.0 mg/dL vs 146.0 ± 45.34 mg/dL, *P* = 0.0078) (Fig. 9e). The number of total Tfh1 cells significantly decreased after GC treatment (37,109 ± 9417 cells/mL vs 22,366 ± 7440 cells/mL, *P* = 0.0391), whereas no obvious change was observed in the number of total Tfh2 cells (28,372 ± 7252 cells/mL vs 28,298 ± 9730 cells/mL, *P* > 0.9999) or the number of total Tfh17 cells (27,072 ± 5646 cells/mL vs 31,770 ± 5467 cells/mL, *P* = 0.7422) (Fig. 9a-c).

Of note, although two patients had normal serum IgG4 in the untreated active phase, the numbers of activated Tfh1 cells and activated Tfh2 cells in the same patients were high compared to HCs. In another patient, disease relapse occurred during GC tapering, and serial analysis showed that the numbers of activated Tfh1 cells and activated Tfh2 cells increased again at the time of relapse.

## Discussion

This study demonstrated that Tfh2 cells, but not Tfh1 or Tfh17 cells, induced naïve B cells to differentiate into plasmablasts and to produce IgG4 in patients with active, untreated IgG4-RD. Of note, IgG4 production by Tfh2 cells from patients with active, untreated IgG4-RD was enhanced compared to those from HCs. Further, activated Tfh2 cells were linked to disease activity in patients with IgG4-RD in the baseline active, untreated phase. Moreover, activated Tfh2 cells decreased with GC treatment and with improvement in disease activity. Interestingly, the number of activated Tfh1 cells was increased in IgG4-RD, and correlated with disease activity, but not with serum IgG4. These results suggest that both activated Tfh1 and Tfh2 cells are the underlying pathological abnormalities playing different roles in IgG4-RD.

Given that the immunoglobulin genes of expanded plasmablasts exhibit somatic hypermutation in patients with IgG4-RD, the expansion of plasmablasts appears to be generated by a T-cell-dependent immune response [15]. Previous studies have assumed that T helper 2 cells and regulatory T cells contribute to IgG4 class-switching in IgG4-RD pathogenesis [35–40], but empirical evidence to show whether these cells actually induce the differentiation of naïve B cells into plasmablasts and the production of IgG4 in patients with IgG4-RD is still lacking. In contrast, Tfh cells are increasingly recognized as CD4^+^ T cells that play an essential role in T-cell-dependent immune response [16, 17], and consist of three subsets (Tfh1, Tfh2, and Tfh17) [19]. Here, our finding that Tfh2 cells, but not Tfh1 and Tfh17 cells, induce the differentiation of naïve B cells into plasmablasts and the production of IgG4 in patients with active, untreated IgG4-RD suggests that Tfh2 cells are pathogenic CD4^+^ T cells that play a pivotal role in the T-cell-dependent immune response in patients with IgG4-RD. To our knowledge, this is the first study to describe the functional role of Tfh2 cells in helping B cells in a vitro assay in patients with IgG4-RD.

We observed that activated Tfh2 cells (CCR7^low^PD-1^high^) were increased in patients with IgG4-RD compared to patients with pSS or MCD, and compared to HCs. Importantly, the expression of CCR7 on T cells is well-known to be generally downregulated when they are activated [26]. Therefore, the low CCR7 expression on Tfh2 cells indicates their recent activation [26]. Meanwhile, the expression of PD-1 on Tfh cells is essential for B cell selection and survival in the germinal centers and for further maturation of B cells into plasmablasts and plasma cells to produce antibodies [41]. Collectively, our finding that activated Tfh2 cells are expanded in patients with active, untreated IgG4-RD suggests that Tfh2 cells are recently activated and functionally important for B cell maturation in IgG4-RD. Our feasible model of the pathogenesis of IgG4-RD is that chronic stimulation by an unknown antigen induces the polarization and activation of Tfh2 cells that are the disease-causing CD4^+^ T cells, which may result in the development of germinal centers at affected sites and the generation of IgG4-secreting plasmablasts and plasma cells. One of the factors that could contribute to the increased activated Tfh2 cells in IgG4-RD may be interferon (IFN)-α. In fact, IFN-α is greatly increased in serum and in the affected tissues in patients with IgG4-related pancreatitis [42]. As a matter of fact, the transcription factor IFN regulatory factor 9 activated by IFN-α is known to bind to the promoter region of the *PD-1* gene and induce *PD-1* mRNA transcription [43, 44].

The establishment of appropriate biomarkers that are linked to disease activity in IgG4-RD is important. The occasional finding of IgG4-seronegative IgG4-RD despite active disease indicates that elevated levels of serum IgG4 are not sufficiently sensitive or specific for disease activity [45, 46]. Meanwhile, the plasmablast count has been recently reported to coincide with both the active phase and disease relapse, and has been suggested to be a useful biomarker of disease activity in IgG4-RD [13–15]. In the present study, we have empirically shown that the number of activated Tfh2 cells correlates with disease activity in the baseline active phase in patients with IgG4-RD. Furthermore, we have demonstrated that the number of activated Tfh2 cells parallels disease activity during GC treatment. These results suggest the potential of activated Tfh2 cells as a biomarker in IgG4-RD. We await corroboration of our results in an analysis of a larger cohort.

Although serum sIL-2R is reported to reflect disease activity in IgG4-RD [31–33], the pathologic significance of the serum level is unclear. Here, we demonstrated that serum sIL-2R is positively correlated with the number of activated Tfh1 cells or activated Tfh2 cells, but not with the number of total Tfh2 cells or total Tfh1 cells, serum CRP, or LDH in patients with IgG4-RD. This suggests that sIL-2R is released from activated Tfh1 and activated Tfh2 cells in IgG4-RD and thus reflects disease activity.

Previous studies report that circulating activated Tfh cells are generated from germinal centers in the spleen or lymph nodes [26]. Hyperplastic germinal centers have been rarely observed in patients with pSS or MCD [47, 48] but have been shown to develop in affected organs in patients with IgG4-RD [18]. Here, we found strong correlation between the number of circulating activated Tfh2 cells and the number of affected organs in patients with IgG4-RD, suggesting that these cells are generated from germinal centers in affected organs in patients with IgG4-RD. Further elucidation of Tfh2 cells in affected tissues would be required for the future.

Because IgG4-RD and MCD are both systemic diseases that present with hypergammaglobulinemia and elevated serum IgG4 [7–12], differentiation between these two diseases is sometimes difficult. The hallmark of the pathogenesis of MCD is considered the hyper interleukin (IL)-6 syndrome, which induces B cell differentiation into plasmablasts and plasma cells, resulting in polyclonal hypergammaglobulinemia [49]. On the other hand, IL-4, IL-5, IL-10, IL-13, IL-21, transforming growth factor (TGF)-β and B cell activating factor (BAFF) are considered the key cytokines in the pathogenesis of IgG4-RD [1–3]. Additionally, in the present study, we showed that the number of total Tfh2 cells was significantly higher in IgG4-RD than in MCD. Furthermore, the number of activated Tfh2 cells was also increased in IgG4-RD compared to MCD. Our findings suggest that IgG4-RD is pathogenetically distinct from MCD from the viewpoint of Tfh2 cell immunology in addition to cytokine profiles. To our knowledge, this is the first study that investigated the differences in Tfh2 cells in IgG4-RD and MCD.

Previous reports have shown that IFN-γ is expressed in local affected lesions and circulating IFN-γ-producing CD4^+^cells are increased in IgG4-RD [37, 50, 51]. Interestingly, in our present study the number of Tfh1 cells and their activated phenotype was increased in patients with IgG4-RD compared to patients with pSS or MCD, or HCs. Although the precise function of Tfh1 cells remains to be elucidated, they are known to express CXCR5 and the transcription factor T-bet, and to produce IFN-γ [19]. Thus, they migrate to the germinal centers that express CXCL13, the ligand for CXCR5, and produce IFN-γ in the local affected lesions of IgG4-RD. On that note, in our present study, the number of activated Tfh1 cells correlated with disease activity, but not with serum IgG4, which was reproduced in our co-culture experiment (Fig. 4). Taken together, both Tfh1 and Tfh2 cells are involved in the pathogenesis of IgG4-RD, while Tfh2 cells are rather important in the process of IgG4 production. Importantly, we have observed patients with active IgG4-RD with normal serum IgG4, suggesting that not only Tfh2 cells but also Tfh1 cells play a role in the pathogenesis of IgG4-RD.

In our previous study [20] we observed positive correlation between serum IL-4, the number of Tfh2 cells, and serum IgG4 in IgG4-RD. Therefore, we measured IL-4 in the in vitro experiments. IL-4 was detectable, but unexpectedly, there was no difference between patients with IgG4-RD and HCs. This may be ascribed to the time point of IL-4 measurements; IL-4 was measured at day 7 but not at a shorter time interval from baseline. Thus, our observation may be the result of IL-4 being partly decreased due to the consumption through the IL-4 receptor of naïve B cells to promote the differentiation of naïve B cells into plasmablasts and the production of IgG4.

Our study demonstrated that Tfh2 cells from patients with active, untreated IgG4-RD induced enhanced production of IgG4 compared to those from HCs. The conceivable mechanisms of this phenomenon are: (1) an increased number of activated Tfh2 cells; (2) involvement of altered co-stimulatory molecules in the process of collaboration between Tfh2 cells and B cells [52]; (3) enhanced cytokine production, such as IL-4, IL-10, IL-13 and IL-21 from Tfh2 cells known for IgG4 class-switching [53]; and (4) an intrinsic alteration of naïve B cells in IgG4-RD patients. Although alteration in naïve B cells is a possibility for the underlying abnormality in IgG4-RD, treatment with an anti-CD20 antibody targeting B cells is known to result in relapse in some patients [54]. Therefore, these possible defects of Tfh2 cells in IgG4-RD suggest that targeting activated Tfh2 cells could lead to the development of novel pharmacological strategies aimed at disrupting the T-B-dependent immune response.

## Conclusions

We have demonstrated that Tfh2 cells, but not Tfh1 or Tfh17 cells, induce the differentiation of naïve B cells into plasmablasts and the production of IgG4 in patients with active, untreated IgG4-RD. Production of IgG4 by Tfh2 cells was enhanced in patients with active, untreated IgG4-RD compared to HCs. In addition, increase in activated Tfh2 cells was linked to disease activity, suggesting that Tfh2 cells and their activated phenotype are involved in the pathogenesis of IgG4-RD. Understanding this T cell subset may aid the monitoring of humoral responses in the context of disease activity and therapeutic responsiveness, and provide a basis to better elucidate the dynamics of pathologic Tfh2 cell activity in IgG4-RD. Moreover, the number of activated Tfh1 cells was also increased in IgG4-RD, correlating with disease activity but not with serum IgG4 level, suggesting the involvement of Tfh1 cells, but not in the process of IgG4 production in patients with IgG4-RD.

## Abbreviations

CRP, C-reactive protein; GC, glucocorticoid; HC, healthy controls; IFN, interferon; IgG4-RD RI, IgG4-related disease responder index; IgG4-RD, IgG4-related disease; LDH, lactate dehydrogenase; MCD, multicentric Castleman disease; pSS, primary Sjögren syndrome; SEB, staphylococcal enterotoxin B; SEM, standard error of the mean; sIL-2R, soluble interleukin-2 receptor; Tfh, follicular helper T

## Additional file

Additional file 1: Figure S1. Flow cytometric analysis of the percentage of circulating follicular helper T (*Tfh*) cells and activated Tfh cells. The percentage of Tfh cells (**A**) and activated Tfh cells (**B**) from patients with IgG4-related disease (*IgG4-RD*) (n = 17), primary Sjögren’s syndrome (*pSS*) (n = 20), multicentric Castleman’s disease (*MCD*) (n = 5), and healthy controls (*HC*) (n = 12). **P* < 0.05; ***P* < 0.0001 for analysis using the Kruskal-Wallis test, followed by group-wise comparisons using the Mann-Whitney *U* test. (TIF 42 kb)
