# Supplementary figures and images for: Enhanced IgG4 production by follicular helper 2 T cells and the involvement of follicular helper 1 T cells in the pathogenesis of IgG4-related disease
Source: Arthritis Res Ther. 2016 Jul 13;18:167. doi: 10.1186/s13075-016-1064-4 (PMC4944254; doi:10.1186/s13075-016-1064-4)

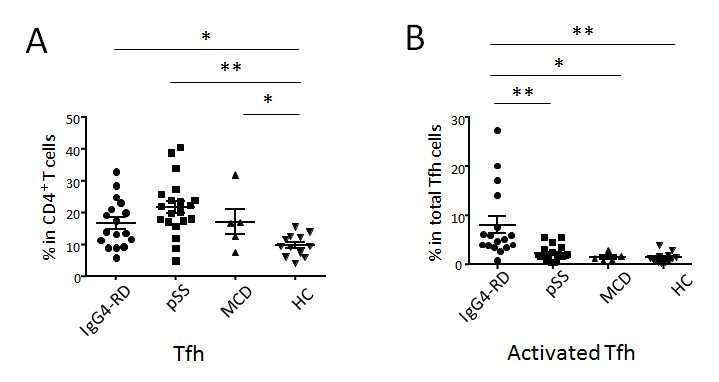

Supplement: Additional file 1: Figure S1. — Flow cytometric analysis of the percentage of circulating follicular helper T (Tfh) cells and activated Tfh cells. The percentage of Tfh cells (A) and activated Tfh cells (B) from patients with IgG4-related disease (IgG4-RD) (n = 17), primary Sjögren’s syndrome (pSS) (n = 20), multicentric Castleman’s disease (MCD) (n = 5), and healthy controls (HC) (n = 12). *P < 0.05; **P < 0.0001 for analysis using the Kruskal-Wallis test, followed by group-wise comparisons using the Mann-Whitney U test. (TIF 42 kb) [file 13075_2016_1064_MOESM1_ESM.tif]
